# Supplementary material for: Co-Infection of Potential Tick-Borne Pathogens of the Order Rickettsiales and Borrelia burgdorferi s. l. and Their Link to Season and Area in Germany
Source: Microorganisms. 2023 Jan 7;11(1):157. doi: 10.3390/microorganisms11010157 (PMC9861244; doi:10.3390/microorganisms11010157)
Supplement: Supplementary file 1 [file microorganisms-11-00157-s001.zip › Supplementary Figure S1+S2.pdf]

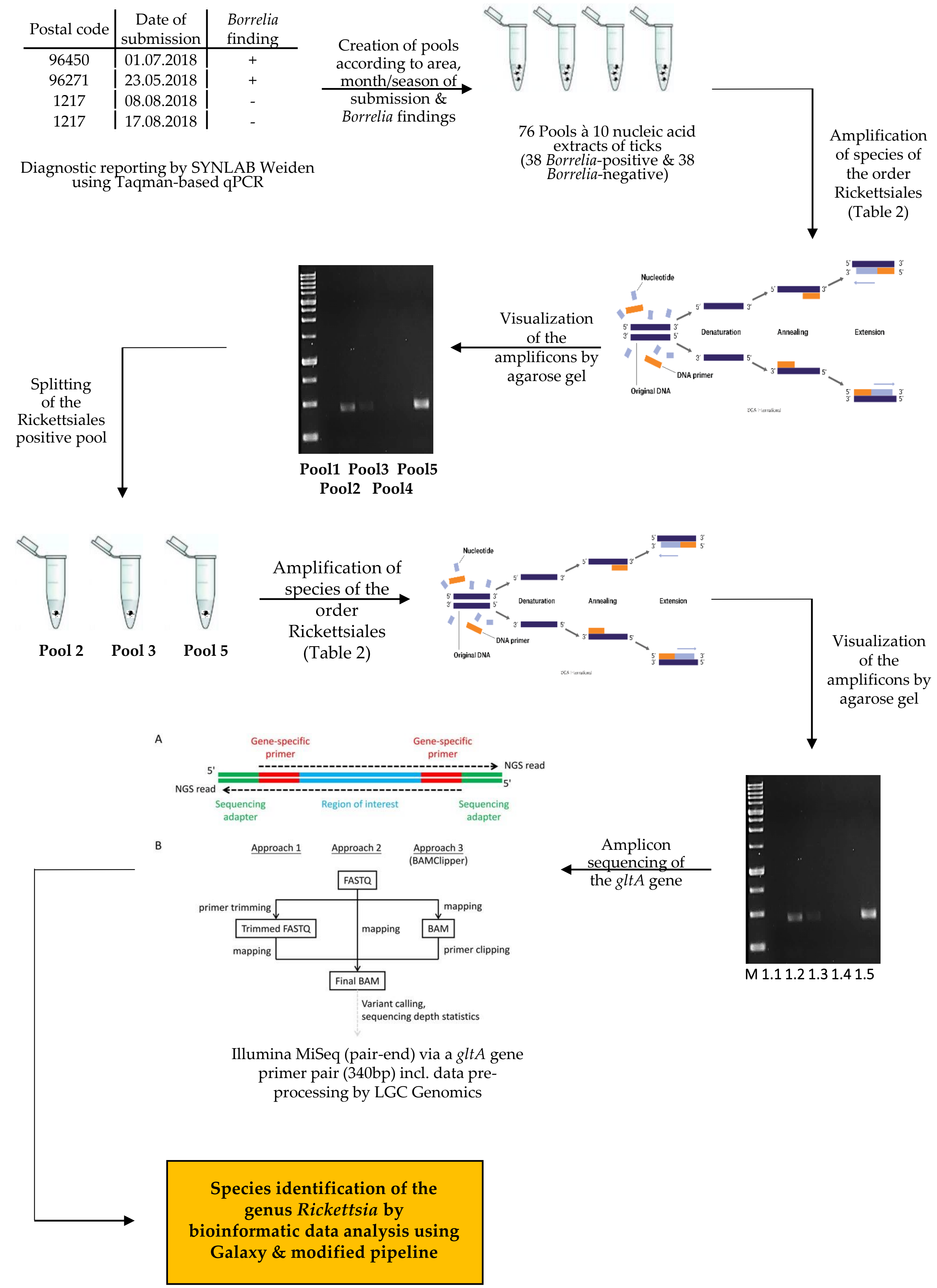

Figure S1. Flowchart of pool assembly from tick nucleic acid extracts to assess tick-associated pathogens of the order Rickettsiales, and subsequent *gltA* gene-based sequencing.

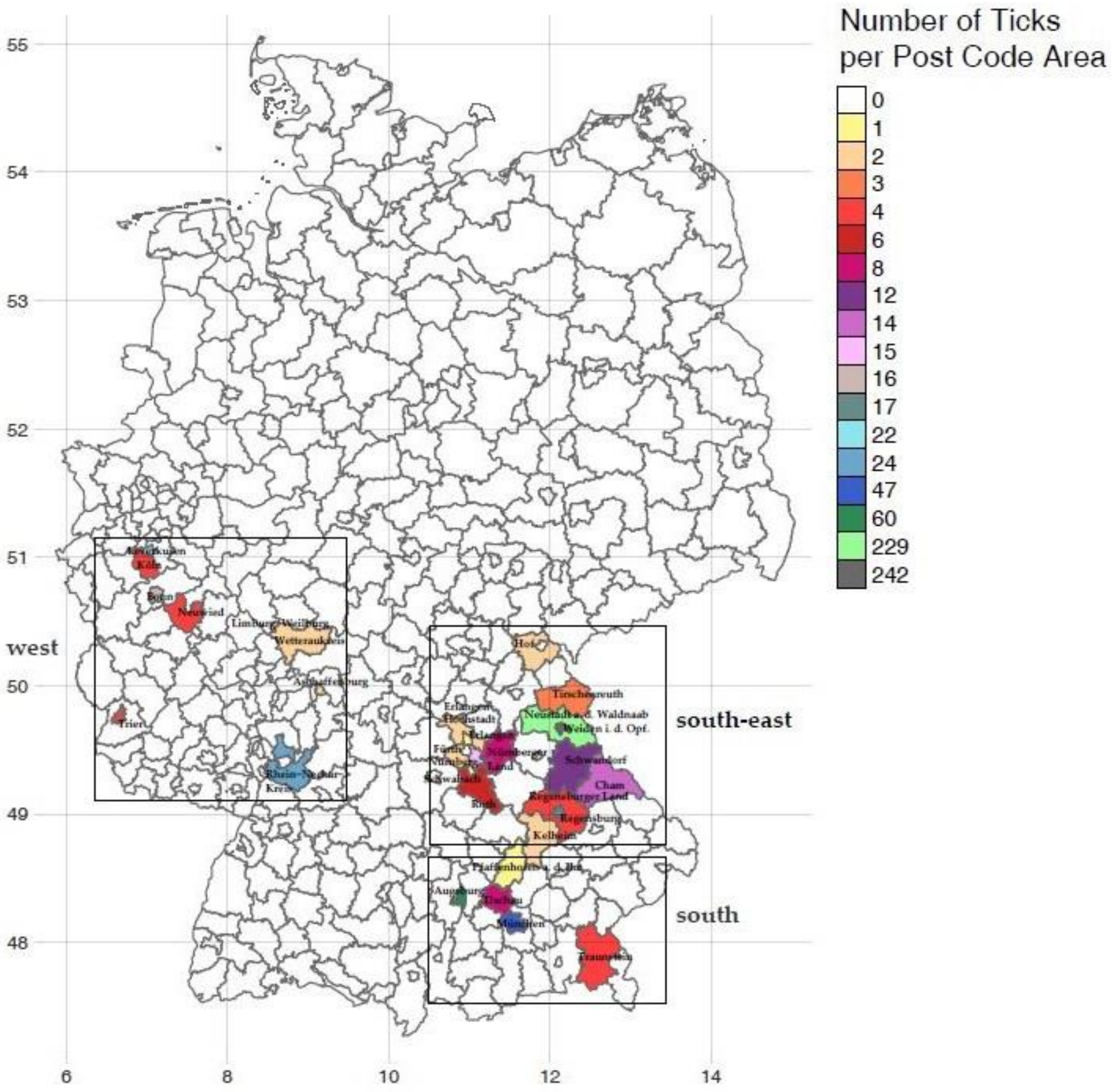

Figure S2. Origin of *I. ricinus* ticks on a map of Germany with identification of the districts and district-free cities for the year 2018. Colors indicate the available tick nucleic acid extracts per post code area in the figure.
